# A Pregnant Woman With Severe Symptomatic Aortic Stenosis: How Should We Treat?

**DOI:** 10.1016/j.jaccas.2023.102205

**Published:** 2024-01-05

**Authors:** Oren Yagel, Roie Alter, Batel Nissan, Donna R. Zwas, Joshua I. Rosenbloom, Ophir Eyal, Mordechai Golomb, David Planer, Gabby Elbaz-Greener

**Affiliations:** a Department of Cardiology, Hadassah Medical Center, The Faculty of Medicine, Hebrew University of Jerusalem, Jerusalem, Israel; b Department of Obstetrics and Gynecology, Hadassah Medical Center, The Faculty of Medicine, Hebrew University of Jerusalem, Jerusalem, Israel; c Department of Nephrology and Hypertension, Hadassah Medical Center, The Faculty of Medicine, Hebrew University of Jerusalem, Jerusalem, Israel

**Keywords:** end-stage kidney disease, high-risk pregnancy, severe aortic stenosis, transcatheter aortic valve replacement

## Abstract

A 31-year-old woman with end-stage kidney disease and with a bicuspid aortic valve presented with acute heart failure in the second trimester of pregnancy. The patient received a diagnosis of severe aortic stenosis and chose to continue the pregnancy against medical advice. Following a multidisciplinary team consultation, she underwent urgent transcatheter aortic valve replacement.

## Graphical abstract

**Figure:**
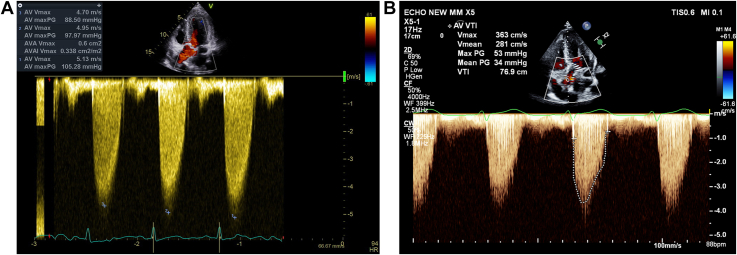

## History of Presentation

We present a case of a 31-year-old woman in her second pregnancy, with end-stage kidney disease (ESKD) and severe aortic stenosis (AS). Early in pregnancy, she was informed about the potential life-threatening risks associated with this pregnancy but made the decision to continue the pregnancy. At the 15th week of gestation, she underwent an indicated cerclage procedure using regional anesthesia, and it was uneventful. At the 22nd week of gestation, she presented with increased dyspnea on exertion and orthopnea while undergoing hemodialysis. On admission, her hemodynamic status was stable, and the physical examination revealed prominent jugular venous distention, a 3/6 systolic ejection murmur, clear lung sounds, a gravid abdomen, and no peripheral edema. She was admitted to the cardiac intensive care unit for further investigation.

**Learning Objectives**

• To understand the management challenges and treatment options for a pregnant patient with severe AS and end-stage kidney failure who is undergoing hemodialysis.
• To recognize the importance of multidisciplinary collaboration in decision making in complex clinical cases, particularly when there is an ethical consideration to respect the patient’s autonomy, which may contradict the recommended medical practice.

## Past Medical History

**Figure 1:**
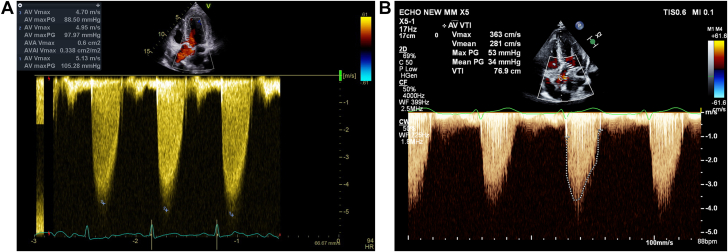
Echocardiogram: Continuous-Wave Doppler Imaging of the Aortic Valve (A) Before intervention. (B) After transcatheter aortic valve replacement. AV = aortic valve; CF = color flow; CW = continuous wave; ECHO = echocardiogram; max = maximum; min = minimum; PG = pressure gradient; V = velocity; VTI = velocity time integral; 2D = 2-dimensional.

The patient has ESKD secondary to nephronophthisis, an autosomal recessive inherited disease. She has been treated with hemodialysis through arteriovenous fistula for the past 7 years. In her previous (first) pregnancy, which occurred during a time when she was undergoing dialysis, she experienced cervical dilation and preterm premature rupture of membranes at the 18th week of gestation, resulting in the termination of the pregnancy followed by dilatation and curettage. Moderate AS in the setting of a bicuspid aortic valve was diagnosed at termination of her previous pregnancy. Echocardiography performed at 6 weeks of the current gestation revealed an aortic valve pressure gradient of 53/34 mm Hg, with a calculated aortic valve area (AVA) of 0.6 mm^2^. The patient was asymptomatic. After the patient opted to proceed with the pregnancy, she was presented at the cardio-obstetrics multidisciplinary team meeting for consideration of empirical valvular intervention at 22 to 25 weeks vs observation. Because the patient was undergoing dialysis daily and was supervised closely, the decision was made to continue close observation. Throughout the pregnancy, the patient underwent dialysis 6 days per week and had regular clinic visits and monthly echocardiography evaluations, which showed AS with a further elevation of gradients, reaching a peak of 105 mm Hg and a mean of 55 mm Hg (Figure 1A), with a calculated AVA of 0.5 cm^2^. At the 22nd week of gestation, the patient was admitted to the hospital with chest discomfort, dyspnea on minimal exertion, and orthopnea.

## Management

**Figure 2:**
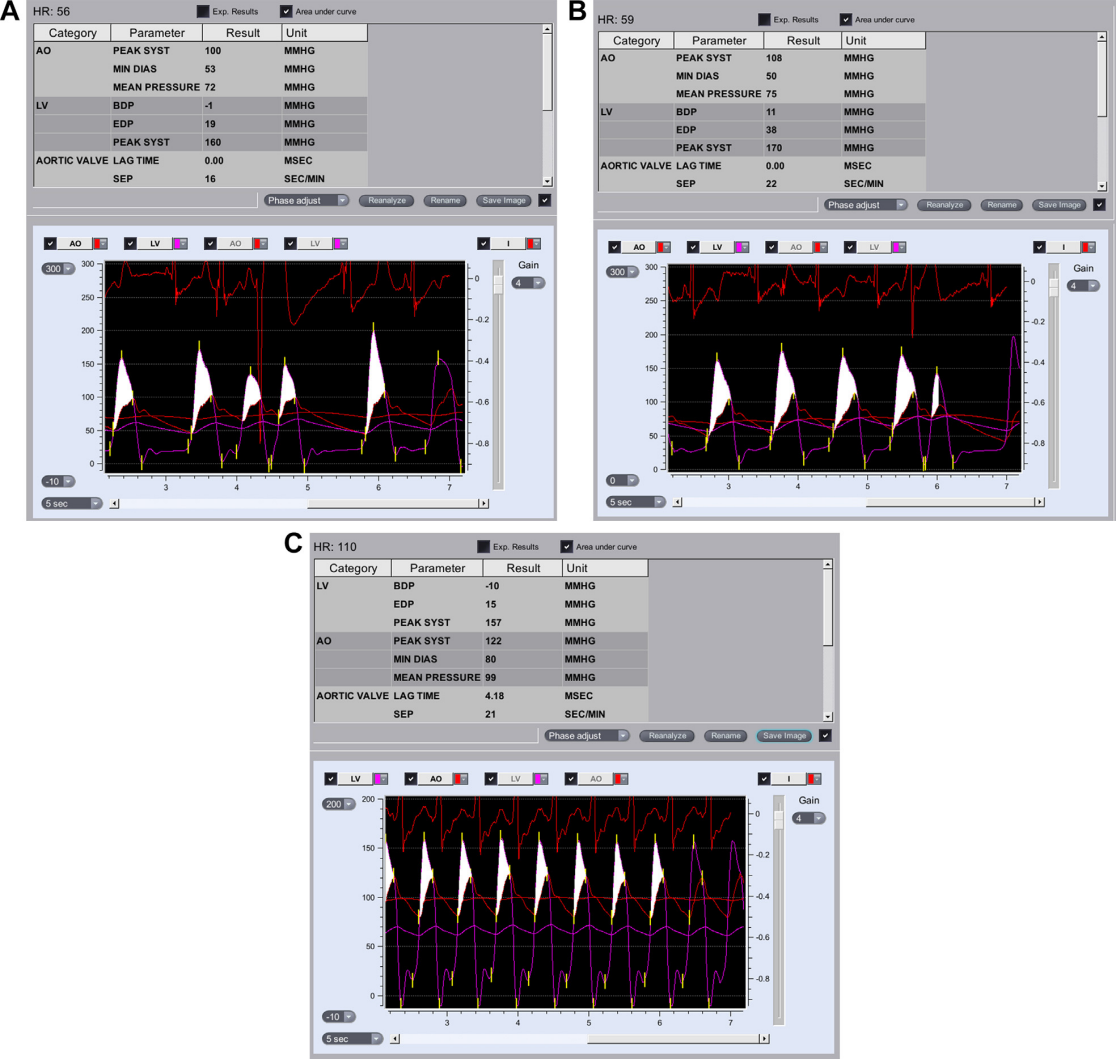
Peak-to-Peak Gradients (A) Before intervention. (B) After balloon aortic valvuloplasty. (C) After transcatheter aortic valve replacement. AO = aorta; BDP = begin diastolic pressure; DIAS = diastolic; EDP = end diastolic pressure; HR = heart rate; LV = left ventricle; MIN = minimum; SEP = systolic ejection period; SYST = systolic.

**Figure 3:**
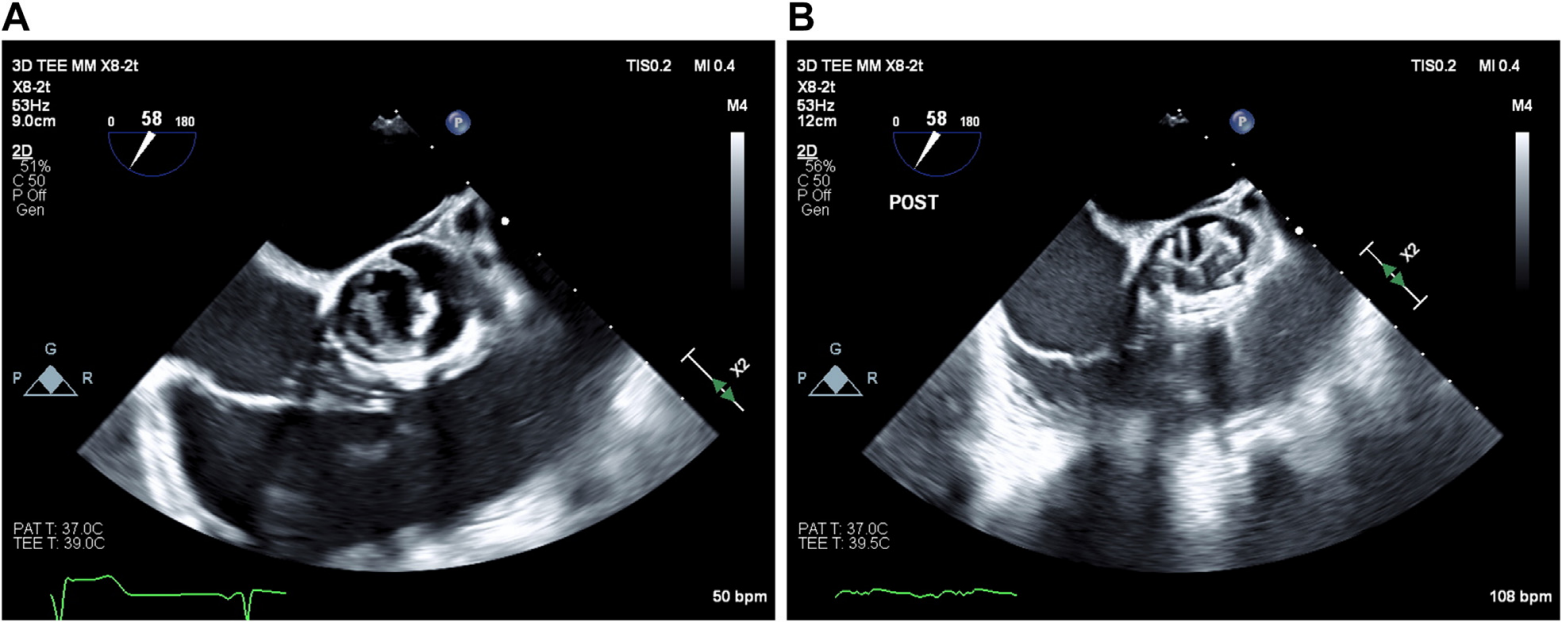
Transesophageal Echocardiogram of the Aortic Valve (A) Before transcatheter aortic valve replacement. (B) Post transcatheter aortic valve replacement. bpm = beats/min; G = general; P = penetration; PAT = patient; R = resolution; T = temperature; TEE = transesophageal echocardiogram; 2D = 2-dimensional.

**Figure 4:**
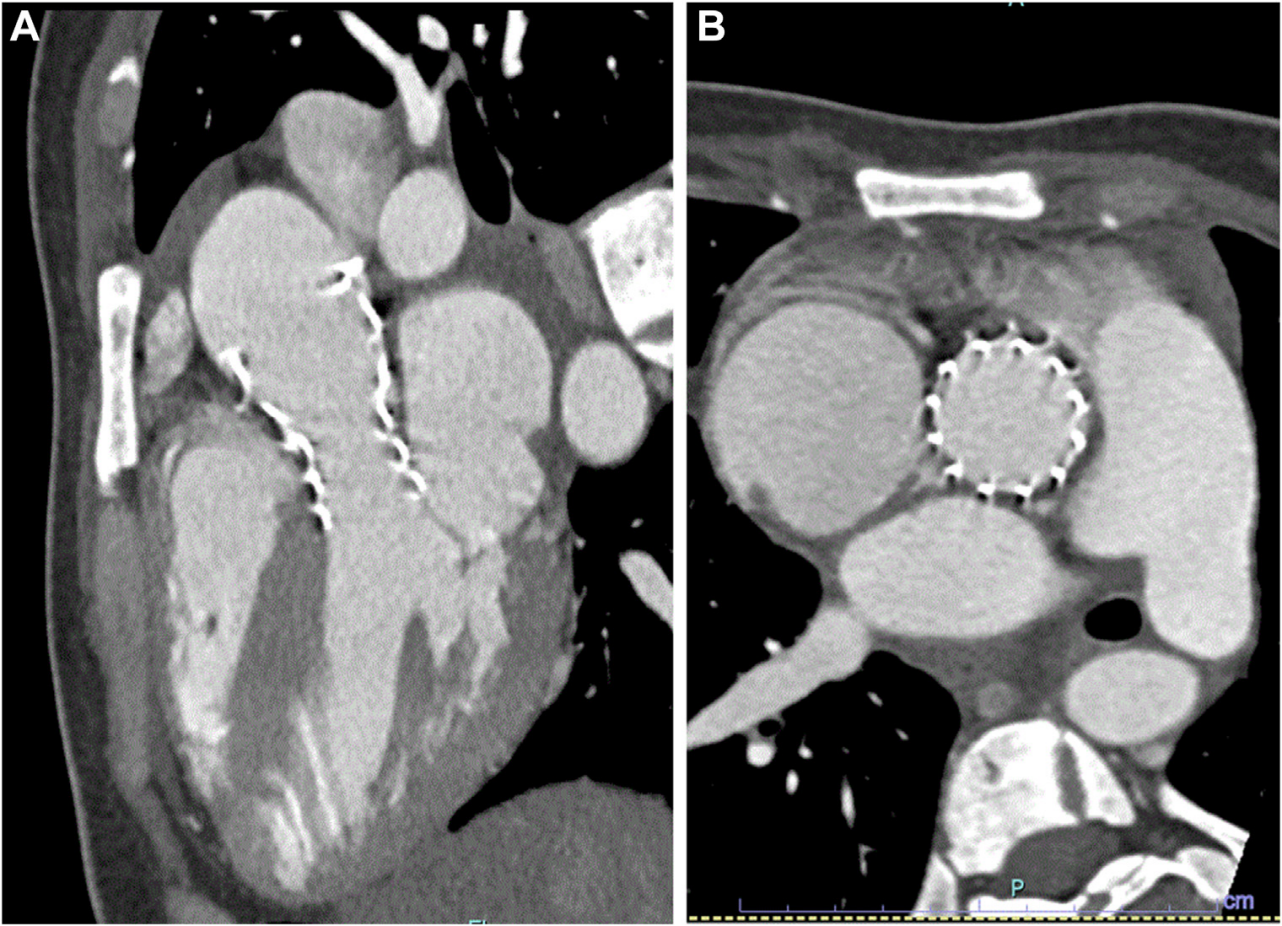
Computed Tomography 6 Months Post-Transcatheter Aortic Valve Replacement: Aortic Valve (A) Long-axis view. (B) Short-axis view.

A multidisciplinary team, including cardiologists, a cardiothoracic surgeon, a high-risk obstetrics specialist, a nephrologist, and anesthesiologists, discussed the treatment options. The offered options included balloon aortic valvuloplasty (BAV), transcatheter aortic valve replacement (TAVR), or surgical aortic valve replacement (SAVR) with a very high risk of pregnancy termination. Given the patient’s symptoms early in the second trimester, the decision was made to intervene. The medical team recommended proceeding with BAV, and the patient underwent the procedure while she was under general anesthesia, with the guidance of transesophageal echocardiography to decrease exposure to radiation and iodine that are used in preprocedure computed tomography (CT) and during the TAVR procedure itself (Videos 1 and 2). Annulus parameters were as follows: perimeter, 60 mm; area, 2.73 cm^2^; and mean diameter, 1.79 cm. Using an 18-mm balloon (VACS III, OSYPKA), there was no improvement in the peak-to-peak gradient post-inflation, measured at 60 mm Hg (Figures 2A and 2B), and new moderate aortic regurgitation developed. Following another ad hoc team discussion concerning the suboptimal results of the BAV, a decision was made to proceed with urgent TAVR. During the positioning of the stiff wire in the left ventricle, the patient experienced ventricular fibrillation, requiring immediate cardioversion and a short cycle of chest compression. Implantation of the TAVR valve continued during resuscitation, with patient stabilization after the valve was fully deployed. An Evolut Pro 23 valve (Medtronic) was implanted successfully without significant perivalvular leak (Figures 3A and 3B), resulting in a peak-to-peak gradient of 35 mm Hg (Figure 2C). Subsequently, a balloon post-dilatation was performed using a 20-mm balloon (VACS III, OSYPKA), thus reducing the peak-to-peak gradient to 20 mm Hg. The patient underwent successful extubation and was admitted back to the intensive cardiac care unit. The day after TAVR, an echocardiogram was performed, which showed peak and mean gradients of 53 mm Hg and 34 mm Hg, respectively (Figure 1B). There was no evidence of fetal distress after the procedure, and findings on targeted ultrasound of the fetal brain were normal. The patient was discharged from the hospital 6 days following the procedure. The patient maintained regular and close follow-ups, with monthly echocardiograms and routine dialysis. During the third trimester, she was hospitalized to enable close observation. In week 34, she developed progressive worsening hypertension and headache. The impression was pre-eclampsia, and the patient was referred for induction of labor. A vacuum delivery was successfully performed at the 35th week of pregnancy without complications, with delivery of a male infant weighing 2,350 g. One month after delivery, an echocardiogram revealed peak and mean gradients of 64 mm Hg and 36 mm Hg, respectively. Further evaluation determined that with occlusion of the fistula, gradients decreased to 20 mm Hg. Six months post delivery, a CT scan confirmed proper positioning of the valve (Figures 4A and 4B). Currently, 9 months later, the patient and the baby are doing well.

## Question 1: What Are the Potential Risks of Continuing With the Pregnancy in a Patient With Severe Aortic Stenosis?

Severe AS in pregnancy is associated with an increased risk of heart failure, cardiac arrhythmias, and rarely death, and it is recommended that severe stenosis be repaired before contemplating pregnancy. If the patient is asymptomatic before pregnancy, AS can be tolerated well during pregnancy.^1^^,^^2^ In cases of moderate and severe AS, there is an increased risk of preterm birth, intrauterine growth restriction, and low birth weight, which can affect approximately 20% to 25% of offspring. These risks are further amplified in cases of severe AS.^2^ In the ROPAC registry (Registry on Pregnancy and Cardiac Disease), of 96 patients with moderate or severe AS, heart failure occurred in 6.7% of patients who were asymptomatic before pregnancy and in 26.3% of symptomatic patients with severe AS, and patients presented with heart failure at a mean gestational time of 27.2 ± 7.5 weeks.^1^ Of patients with severe AS, more than 42% were hospitalized during admission. Two patients underwent aortic valve interventions during pregnancy. Risk was associated with the severity of stenosis and the extent of the transaortic gradient. In all cases, the presence of moderate or severe AS warrants careful individualized follow by a cardio-obstetric heart team.

## Question 2: What Are the Indications for Balloon Valvuloplasty vs TAVR or SAVR during pregnancy?

Given the absence of large, randomized studies on severe AS during pregnancy, the treatment approach is primarily guided by expert opinion. BAV is considered the preferred initial treatment for symptomatic severe AS.^1^ BAV has demonstrated a lower complication rate compared with TAVR.^3^ The European Heart Society guidelines for managing cardiovascular diseases during pregnancy acknowledge TAVR as a promising treatment option, but its application is still constrained by limited experience and data availability.^4^ SAVR performed during pregnancy carries an increased, yet manageable, risk for the mother (3%-7%). However, it poses a significantly high risk to the fetus. The estimated risk of fetal loss during SAVR is approximately 20%.^5^

## Question 3: What Is the Impact of End-Stage Kidney Disease on Pregnancy and on the Progression of AS?

ESKD significantly increases the risk of adverse maternal and perinatal outcomes. Women undergoing dialysis are at significantly increased risk of pre-eclampsia and increased rates of premature delivery, reported at 40% to 50%.^6^ A large meta-analysis found that the median gestational age for all live births was 33 weeks (range: 26-39 weeks), and 32% (49 of 154) of infants were born small for gestational age.^7^ There are increased rates of cesarean delivery, peripartum transfusion, and overall maternal morbidity and mortality. In this patient, dialysis complicated the assessment of heart failure because clinical manifestations of volume overload could be masked by dialysis, and the measurement of natriuretic peptides may lead to inconsistent results. The prevalence of significant AS is higher in hemodialysis recipients over the age of 65 years, with a rate of 3.3%, compared with 1% to 2% in the general population. Additionally, the occurrence of severe AS is uncommon in those under the age of 50 years, but it increases to 3% among patients with ESKD in the same age group. Patients with ESKD who are undergoing hemodialysis and who develop severe AS experience a worse prognosis compared with patients with severe AS who are not undergoing hemodialysis.^8^

## Question 4: What Is the Impact of Arteriovenous Fistula on the Hemodynamics of Patients With Severe AS?

There are 2 effects of arteriovenous fistula on patients with severe AS. First, the presence of an arteriovenous fistula can complicate the assessment of AS severity. Cardiac output plays a significant role in determining the transaortic valve gradient in AS. In patients with ESKD and suspected severe AS, manually compressing the arteriovenous fistula resulted in a decrease of the mean transaortic valve gradient from 45 mm Hg to 30 mm Hg in 1 report.^9^ In our case, manually compressing the arteriovenous fistula led to a decrease in the mean transaortic valve gradient from 36 mm Hg to 20 mm Hg, thus aligning with findings in other reports. Second, the increase in cardiac output associated with arteriovenous fistula creation can lead to acute or subacute decompensation in patients with significant AS who were previously asymptomatic or had minimal symptoms.^8^

## Question 5: What Is the Optimal Timing for Intervention in Severe AS During Pregnancy?

The highest risk of hemodynamic compromise and heart failure occurs during the second and third trimesters, during labor and delivery, and in the 24 to 72 hours following delivery, times coinciding with the peak of cardiac output.^10^ Fetal mortality following maternal cardiac surgery is notably high, particularly during the first and second trimesters. Performing a cesarean delivery before cardiac surgery, particularly in the third trimester, is an independent factor associated with reduced fetal mortality in cases where cardiac surgery is required.

## Funding Support and Author Disclosures

The authors have reported that they have no relationships relevant to the contents of this paper to disclose.

## Appendix

Video 1 Transesophageal Echocardiogram, Short-Axis View: Bicuspid Aortic Valve, Severe AS

Video 2 Transesophageal Echocardiogram, Long-Axis View: Bicuspid Aortic Valve, Severe AS
